# Is MOOC really effective? Exploring the outcomes of MOOC adoption and its influencing factors in a higher educational institution in China

**DOI:** 10.1371/journal.pone.0317701

**Published:** 2025-02-24

**Authors:** Hao Huang, Dandan Qi

**Affiliations:** 1 Academic Affairs Office, Chongqing Business Vocational College, Chongqing, China; 2 School of Architecture and Civil Engineering, Chongqing Business Vocational College, Chongqing, China; Xi'an Jiaotong-Liverpool University, CHINA

## Abstract

Massive Open Online Course (MOOC) has gained widespread adoption across diverse educational domains and plays a crucial role in advancing educational equality. Nevertheless, skepticism surrounds the effectiveness of MOOC due to their notably low completion rates. To explore the outcomes of MOOC adoption in higher education and improve its application efficiency, this study compares MOOC with traditional course in terms of mean score and pass rate. The study examines the factors influencing MOOC performance within the context of higher education, utilizing the method of Partial Least Squares-Structural Equation Modeling (PLS-SEM). This study analyzed MOOC learning data from a college over a period of six years and a total of 4,282 Chinese college students participated in this study. The factor of learning environment was proposed for the first time, and it was proved to have a significant impact on learning behavior and MOOC performance in higher education. The results reveal that 1) MOOC has a lower pass rate than traditional course (including both compulsory and selective course); 2) MOOC has a lower mean score than selective course only; 3) we did not find a significant difference between MOOC and compulsory course in terms of the mean score; 4) Learning behavior, learning motivation, perceived value, learning environment, previous experience and self-regulation have significant and positive influences on MOOC performance in higher education. The study provides valuable insights that college administrators should pay attention to students’ learning environment, learning motivation and other factors while actively introducing MOOC.

## 1. Introduction

The term of Massive Open Online Course (MOOC) was coined in 2008 by Canadian academics Bryan Alexander and Dave Cormier to describe the online course offered by Siemens and Downs [1]. In recent years, the proliferation of information technology and communication media has catalyzed the swift expansion of MOOC, attracting the engagement of esteemed educational institutions on a global scale. In contrast to the conventional campus-based educational models, MOOC is accessible to a broader demographic of learners who possess internet connectivity, irrespective of enrollment prerequisites [2–4]. There has been a discernible upsurge in the participation of individuals within the MOOC learning. During the outbreak of the COVID-19 pandemic in 2020, students were confined to their homes, making MOOCs an essential teaching and learning method in numerous countries [5]. Data collected by Class Central (www.class-central.com) showed that Coursera, the preeminent provider of MOOC, witnessed a significant influx of 24 million new learners, thereby expanding its global learner constituency to a total of 142 million individuals. This marked the most substantial annual increase in new learners since the advent of the COVID-19 pandemic in 2020, which had catalyzed an augmented appetite for online educational initiatives [6]. MOOC use a variety of interactive tools and multimedia resources, such as video lectures, online discussions, real-time Q&A, and automated assessments, which enrich the teaching methods and learning experience. Therefore, MOOC has been widely used in various types of education, especially in higher education [7–9]. In China, MOOC plays a pivotal role in alleviating the insufficiency of educational resources and it has emerged as a significant pedagogical tool within the educational landscape [10,11]. The Chinese government actively promotes the introduction of MOOC as a supplement to daily teaching and many Chinese universities offer a wide range of MOOC courses for college students [8,12,13]. As the largest higher education system in the world, China presents a distinct environment for MOOC research [14].

Many scholars have studied the outcomes of MOOC adoption, and explored the influencing factors of MOOC performance in higher education [15–17]. However, their research results have not been exactly the same. Some studies have found that MOOC promoted learning performance including students’ learning behaviors and final outcomes [18,19]. Other studies have shown that the effect of MOOC fell to meet expectations, mainly manifested in low learning completion rates [20,21]. Previous research on MOOC has not been comprehensive enough, as most existing studies focus on a micro-level analysis of a specific MOOC course [22,23]. The research on the overall effectiveness of MOOC, as a type of course integrated into the regular curriculum in higher education, is insufficient. As a consequence, stakeholders within educational institutions grapple with a lack of empirical data needed to inform strategic management decisions.

In order to fill this gap, this study aims to analyze the outcomes of MOOC adoption for the higher education institute comparing with traditional course and verify the influencing factors of MOOC performance in order to provide implications for instructors and administrators in colleges. Therefore, the research questions of this study are:

1. Comparing with traditional courses, what are the outcomes of MOOC adoption in higher education?
2. What are the factors influencing the MOOC performance from the perspective of education administration in higher education?

## 2. Literature review

### 2.1 MOOC effectiveness

Higher education institutions, as the primary suppliers of premium MOOC content, are proactively incorporating MOOC as additional resource to complement their conventional teaching methods. Over the last few years, studies about MOOC and its adoption in higher education have increased exponentially. Research on the MOOC design and adoption showed that MOOC was proved to be a beneficial and valuable online resource for both lecturers and students in the teaching and learning of different subjects [18,19,24]. From a case study of 1,515 students in a higher education institution in Europe, Despujol, Castañeda [15] found MOOC could improve professional careers and training for the students. In some specific areas, such as medicine, MOOC has also been proved to have positive teaching effects. The Polytechnic University of Milan in Italy employed MOOC as foundational courses and discovered that they positively impacted the development of essential competencies in physics [25]. Gao, Yang [26] from China conducted a meta-analysis to compare the impact of MOOC and traditional teaching methods in medical education and found that the final test scores of participants in the MOOC group were significantly higher than those in the control group, indicating that MOOC is suitable for medical education.

### 2.2 Challenges in MOOC adoption

While supporters of the MOOC movement argue that MOOC can provide significant educational advantages to both academic institutions and their students, mixed results emerged from the literature in China and other countries of the world. The consistently high attrition rate among students in online courses is widely recognized as a significant issue and a cause for concern among educational professionals [20,21,27]. By analyzing data from 298 college students, Patra, Dutta [16] found that although MOOC had been applied in university teaching, their course selection and completion rates were still very low. A study conducted on a MOOC offered by a Spanish university indicated that MOOC learners had problems of collaborative activities and insufficient time which might lead to dropout [23]. In addition to low registration and completion rates, poor learning behavior is another prominent issue in MOOC learning. Analyzing data of three MOOC courses at Yuan Ze University, Tseng, Tsao [17] found that the average numbers of logging and video watching in these courses were relatively low.

### 2.3 Factors influencing MOOC performance

With the surge in the number of MOOC users, understanding and improving learners’ learning performance effectively has become an urgent issue in this field. In an effort to address the issue of low completion rates, researchers have dedicated significant time and resources to identifying the factors that contribute to MOOC performance.

#### 2.3.1 Learning behavior.

Learning behavior refers to various activities and interaction patterns that learners display when participating in MOOC learning. These behaviors include watching video lectures, completing assignments, participating in online discussions, reading course materials, social interaction and making study plans. Compared with traditional course, the learning behavior in MOOC is characterized by its fragmented nature, lack of planning, and being assigned a low priority [28]. Learning behaviors provide educational researchers with rich data for analysis [29]. Jin [30] made a MOOC student attrition prediction model based on learning behavior characteristics, which could significantly improve the prediction of MOOC performance. Li, Du [31] analyzed the learning engagement, time organization, content access sequence and activity participation patterns of MOOC learners with different achievement levels, and found that successful learners need to complete a certain level of tasks as a basic guarantee for passing the course. Through these forms of assessment, learners can test their understanding of course content and adjust their learning strategies in time. Effective time management helps students maintain the continuity of learning, thereby improving learning efficiency and performance. Wang, Dong [10] constructed a theoretical model based on the Theory of Planned Behavior. The research results show that learning behavior has a significant impact on learning performance. Learners who are able to set clear learning goals and track their progress are more likely to complete courses and achieve good grades. Communicating with other learners can provide additional learning resources and perspectives, helping learners understand course content from different perspectives. Wang, Guo [32] revealed the impact of the phenomenon of group rational behavior in online learning on course selection results. By optimizing and adjusting learning behaviors, students can absorb knowledge, improve skills, and ultimately improve learning performance effectively. Therefore, we propose the following hypothesis regarding the learning behavior:

*H1: Learning behavior has a significant positive impact on MOOC performance in higher education.*

#### 2.3.2 Learning motivation.

Learning motivation refers to the internal psychological factors and external conditions that drive learners to participate in MOOC learning activities. Learning motivation can be classified and understood from different perspectives, mainly including intrinsic motivation, extrinsic motivation and enhanced motivation [28,33,34]. From the comparison of MOOC and traditional course, the differences on requirement and selection lead to different learning motivation. Learning motivation is an important factor that affects learners’ initiation, maintenance and completion of learning tasks [3]. Research from de Barba, Kennedy [35] showed that learning motivation affected performance through students’ participation in courses. Different types of motivation also lead to different learning behaviors. Research results from Chi [36] showed that motivation played a mediating role in the influencing factors of learning participation. Students with strong intrinsic motivation are more likely to choose challenging tasks, invest more time and energy in learning, and show higher autonomy and innovation in the learning process. Lee and Song [37] explored how learners’ motivational variables affected their learning persistence, and the research results showed that motivation and self-efficacy had a significant positive impact on learning persistence. Zhu and Doo [38] explored the correlation between motivation, self-monitoring, self-management, and the learning strategies employed by MOOC participants. The results indicated that motivation positively influences self-monitoring, self-management, and the adoption of learning strategies, highlighting the importance of focusing on the motivational aspects of learners. Therefore, the positive relationships between learning motivation and MOOC performance, learning motivation and learning behavior in higher education are proposed.

*H2a: Learning motivation has a significant positive impact on MOOC learning behavior.*

*H2b: Learning motivation has a significant positive impact on MOOC performance.*

#### 2.3.3 Perceived value.

Perceived value refers to learners’ subjective evaluation and perception of the value and benefits of MOOC learning. Perceived value has a significant impact on learners’ behavior and decision-making. If learners believe that a course has high perceived value, they are more likely to register for this course, actively participate in all aspects of the learning activities, persist in completing the course, and recommend the course to others in the future [4,39]. When learners believe that the course is valuable to them, they are more likely to adopt autonomous learning behaviors, such as setting learning goals, planning learning schedules, and finding and utilizing additional learning resources. Padilha, Machado [40] conducted research on nurses’ assessments regarding the ease of use, utility, and their willingness to engage with MOOC. The findings revealed a strong positive correlation between the perceived usefulness of MOOC and the nurses’ intention to persist in utilizing it. When learners have high perceived value, they are more likely to devote more time, which usually results in better learning performance. Learners with high perceived value are also more likely to persist through the course. The study from Chen and Chen [41] documented the facilitation process, key influencing factors, and students’ perceived gains in a six-week MOOC study group. The results showed that students with high perceived value were more willing to try new features, new courses and new learning strategies of MOOC. Through meta-analysis, Zhang and Li [42] determined that perceived value played an important role in MOOC learners’ continued use intention and previous learning experience had moderating effects on perceived value. Consequently, we formulate the following hypothesis:

*H3a: Perceived value has a significant positive impact on MOOC learning behavior.*

*H3b: Perceived value has a significant positive impact on MOOC performance.*

#### 2.3.4 Learning environment.

Learning environment provides learners with physical and technical support for learning. The learning environment, encompassing both the locations and the tools used for learning, constitutes significant external factors within the MOOC learning process. Learning location refers to the physical and psychological place where students are located during the learning process. The physical place includes hardware facilities such as classrooms, libraries, and dormitories, while the psychological place involves factors such as the learning atmosphere, teacher-student relationship, and classmate relationship [3]. Learning tools refer to tools that assist students in acquiring knowledge and improving learning efficiency and effectiveness during the learning process. They can be physical tools (such as textbooks, notebooks, experimental equipment, etc.) and digital tools (such as e-readers, online learning platforms, educational software). A good learning environment can motivate students to be diligent and improve learning efficiency [43]. A quiet and comfortable learning environment helps learners concentrate and reduce external interference, thereby improving learning performance. A good learning environment can also encourage learners to devote themselves to learning for a longer period of time and increase the frequency and persistence of learning behaviors [33,44]. The resources provided by the learning location, such as libraries, laboratories or network facilities, have a direct impact on learning performance. Learners can easily access rich learning resources, which help them better understand and master course content, and also increase their participation in course discussions and activities [45,46]. Consequently, we formulate the following hypothesis:

*H4a: Learning environment has a significant positive impact on MOOC learning behavior.*

*H4b: Learning environment has a significant positive impact on MOOC performance.*

#### 2.3.5 Previous experience.

Previous experience in this study refers to the learner’s historical MOOC learning situation, including the use of the platform, interactions between teachers and students, challenges and achievements that learners experienced during their participation in the MOOC learning before. A rich and positive learning experience can not only improve learners’ knowledge and skills, but also enhance their motivation and self-confidence, and promote personal growth and career development. Li and Wan [47] found that learners with more previous learning experience encountered fewer difficulties in the learning process and had higher course completion rates. Learners with rich learning experience are more likely to adapt to the same type of course. Past learning experiences can develop learners’ autonomous learning abilities, which enable learners to independently plan learning paths, select learning resources, and monitor their own learning progress. Li [48] examined the impact of learning experience and other factors on goal setting. The results showed that learning experience had an impact on thinking, behavior, motivation and choice in the learning process. MOOC is a relatively new way of learning and students who are not familiar with network technology often encounter various problems during the first learning process. The learning methods and strategies that learners have developed in their past studies will directly affect their learning efficiency and effectiveness in MOOC courses. Luik, Feklistova [49] studied the demographic and social background characteristics of MOOC participants and found that the perceived difficulty of MOOCs was related to the distribution of education levels. The results showed that previous experience levels affected the completion of MOOCs. Therefore, the positive relationships between previous experience and MOOC performance, previous experience and learning behavior are proposed.

*H5a: Previous experience has a significant positive impact on MOOC learning behavior.*

*H5b: Previous experience has a significant positive impact on MOOC performance.*

#### 2.3.6 Self-regulation.

Self-regulation pertains to an individual’s capability to efficiently oversee and direct their own actions, emotions, and thought processes when confronted with a range of alluring distractions, stressors, and obstacles. This skill enables individuals to withstand immediate enticements, surmount challenges, and maintain adherence to a predetermined course of action [50]. Self- regulation is important in the field of learning and it can also help students better cope with setbacks and difficulties in learning in order to maintain a positive learning attitude and motivation [51]. Self-regulation is especially important in MOOC learning environments because MOOC often offer greater flexibility and autonomy than traditional face-to-face courses. Md Zalli, Nordin [52] did a study to examine the role of self-regulated learning and found it was a significant factor influencing learners’ satisfaction in a Malaysia MOOC. learners with strong self-regulation show higher persistence in learning behaviors. Zhu and Doo [38] investigated the relationship between motivation, self-regulation and the learning strategies used by MOOC learners. The findings indicated that self-regulation had a positive impact on learning strategies, highlighting the critical need to enhance self-regulation skills to further promote learning strategies. Students with strong self-regulation abilities generally perform better than students with weak self-regulation abilities in various subject areas. Reparaz, Aznárez-Sanado [53] assessed the difference in self-regulation between MOOC course completers and non-completers. The study found that students who completed the course were better at self-regulation and showed higher levels of engagement with MOOC content. Self-regulation learners are more likely to actively participate in various learning activities in the course, because they can effectively organize their learning time and tasks. Sambe, Bouchet [54] and [55] proposed conceptual frameworks that can improve the quality of learning in MOOC and increase satisfaction by promoting self-regulated learning in MOOC. Therefore, the positive relationships between self-regulation and MOOC performance, self-regulation and learning behavior in higher education are proposed.

*H6a: Self-regulation ability has a significant positive impact on MOOC learning behavior.*

*H6b: Self-regulation ability has a significant positive impact on MOOC performance.*

#### 2.3.7 Other factors.

Gamage, Fernando [56] found that learners’ perceptions and choices of MOOC were affected by interactivity, collaboration, etc. Aldowah, Al-Samarraie [57] conducted research on the factors influencing MOOC dropout and found that timely intervention can improve completion rates. Zhu, Sabir [58] discussed how to address the cultural diversity of learners in MOOC design and delivery. They recommended that MOOC design should consider cultural diversity and meet the needs of learners from different cultural backgrounds through strategies such as providing subtitles, adjusting teaching content and methods and encouraging interaction among learners.

Despite the findings above, these studies have several limitations. First, most of the existing studies have investigated MOOC adoption and factor analysis only for a specific subject or course, lacking a holistic analysis from the administrative level [22,23]. Second, the conclusions from existing studies are from just one run of MOOC learning and few literature have examined the outcomes of MOOC using longitudinal studies [9,59]. The methodology and design of this study are used to fill these gaps.

## 3. Method

### 3.1 Research design

The research was carried out at a local college in China, a full-time public residential institution with an enrollment of over 15,000 students. In this college, the types of courses include compulsory course (CC), selective course (SC) and MOOC. CC and SC are traditional courses while MOOC is a new type of course launched in 2017 in this college. The comparison of characteristics for each type of course is shown in Table 1. To fulfill graduation requirements, students are required to finish all CC and earn eight credits from either SC or MOOC. Within a single semester, students are permitted to select only one MOOC from the Chaoxing MOOC platform. Upon successful completion of MOOC, students are awarded one credit.

**Table 1:** Comparison of each type of course.

Category	Traditional Course		MOOC
	Compulsory Course	Selective Course	
Course Selection	CC is chosen by the college	instructor provides an SC list and the student chooses one	student choose MOOC from the platform
Requirement	must complete all CC	get enough credits	get enough credits
Teaching method	face-to-face teaching	face-to-face teaching	video and online resource
Time management	scheduled	scheduled	self-paced with deadline
Learning tool	Supported by college	Supported by instructor	Not supported
Location	classroom	classroom	online
Example	Advertising creativity and planning, Fundamentals of Accounting, Procurement & Supply Management, etc.	Speeches and recitations, Music appreciation, Traditional Chinese Culture, etc.	Innovative thinking training, Food safety and daily diet, Emotion management, etc.

**Fig 1:**
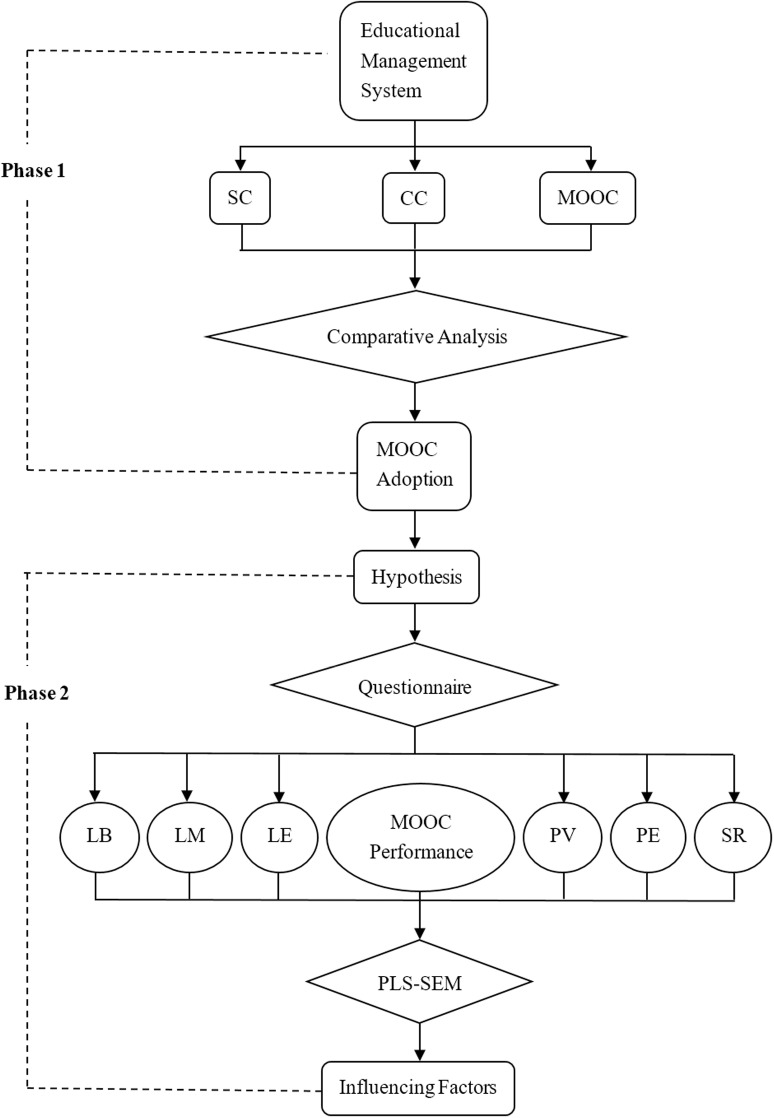
Analysis process.

According to the research questions, this study consisted of two phases. In the first phase, the comparative analysis [60] of the outcomes from MOOC and traditional courses was carried out to explore the difference in terms of mean score and pass rate for the data of past six years. In the second phase, based on the characteristics of MOOC, the hypothesis and conceptual model were established and verified by PLS-SEM to explore the influencing factors. The main reason for choosing this technique is that PLS-SEM enables the analysis of relationships between theoretical constructs and evaluates the model’s validity and reliability. In addition, it is suitable for testing hypotheses in studies with multiple and complex relationships [61]. The flow chart of analysis process is shown in Fig 1.

### 3.2 Phase 1

#### 3.2.1 Sample.

We used the course data generated in the educational management system from 2017 to 2022 including the COVID-19 pandemic year on 15^th^ October 2022, a total of 12 semesters, for comparative analysis. The process of dataset anonymization was conducted. Full names were replaced with identifiers that could not be traced back to the individual. Residential and email addresses were completely removed. The data cleansing was done to filter out the missed fields. To answer question 1, the courses in the system were labelled CC, SC and MOOC. The demographic information of students for comparative analysis is shown in Table 2.

**Table 2:** Demographic information of participants in phase 1.

Category	Group	N	*%*
Gender	Male	8,371	36%
	Female	14,866	64%
Faculty	Accounting	7,976	34%
	Management	5,317	23%
	Information & Technology	6,652	29%
	Tourism & Catering	3,128	13%
	N/A*	164	1%
Total		23,237	

*Some students changed their faculties during their study, and they are marked as N/A.

#### 3.2.2 Data analysis procedure and techniques.

The analysis proceeded in two steps. First, we obtained all the course grades for these 12 semesters from the educational management system. Through the descriptive statistical analysis in SPSS, we got course pass rates and mean scores for CC, SC and MOOC in each semester. In the second step, after verifying the assumption of normality (p >  0.05), as shown in Table 3, paired T-test [60,62] was conducted to assess the pass rate for all three paired courses (CC-SC, p =  0.089; CC-MOOC, p =  0.179; SC-MOOC, p =  0.560) and mean score for paired CC-SC (p =  0.338). Wilcoxon signed-rank test was used to analyze the mean score of paired CC-MOOC (p =  0.044) and SC-MOOC (p =  0.002), because the data did not meet the normality assumption (p <  0.05) [63].

**Table 3:** Shapiro-Wilk Test of Normality.

Paired	df	Pass rate		Mean score	
		Statistic	Sig.	Statistic	Sig.
CC-SC	11	0.875	0.089	0.922	0.338
CC-MOOC	11	0.899	0.179	0.851	0.044
SC-MOOC	11	0.943	0.560	0.753	0.002

### 3.3 Phase 2

From the literature reviewed and comparison results of characteristics for each type of course, we developed a research model with MOOC performance and its influencing factors in the context of higher education from the perspective of education administration. It is posited that a variety of factors can impact MOOC performance, including learning behavior, motivation, perceived value, the learning environment, prior experience, and self-regulation [3,4,9,28].

#### 3.3.1 Research model.

**Fig 2:**
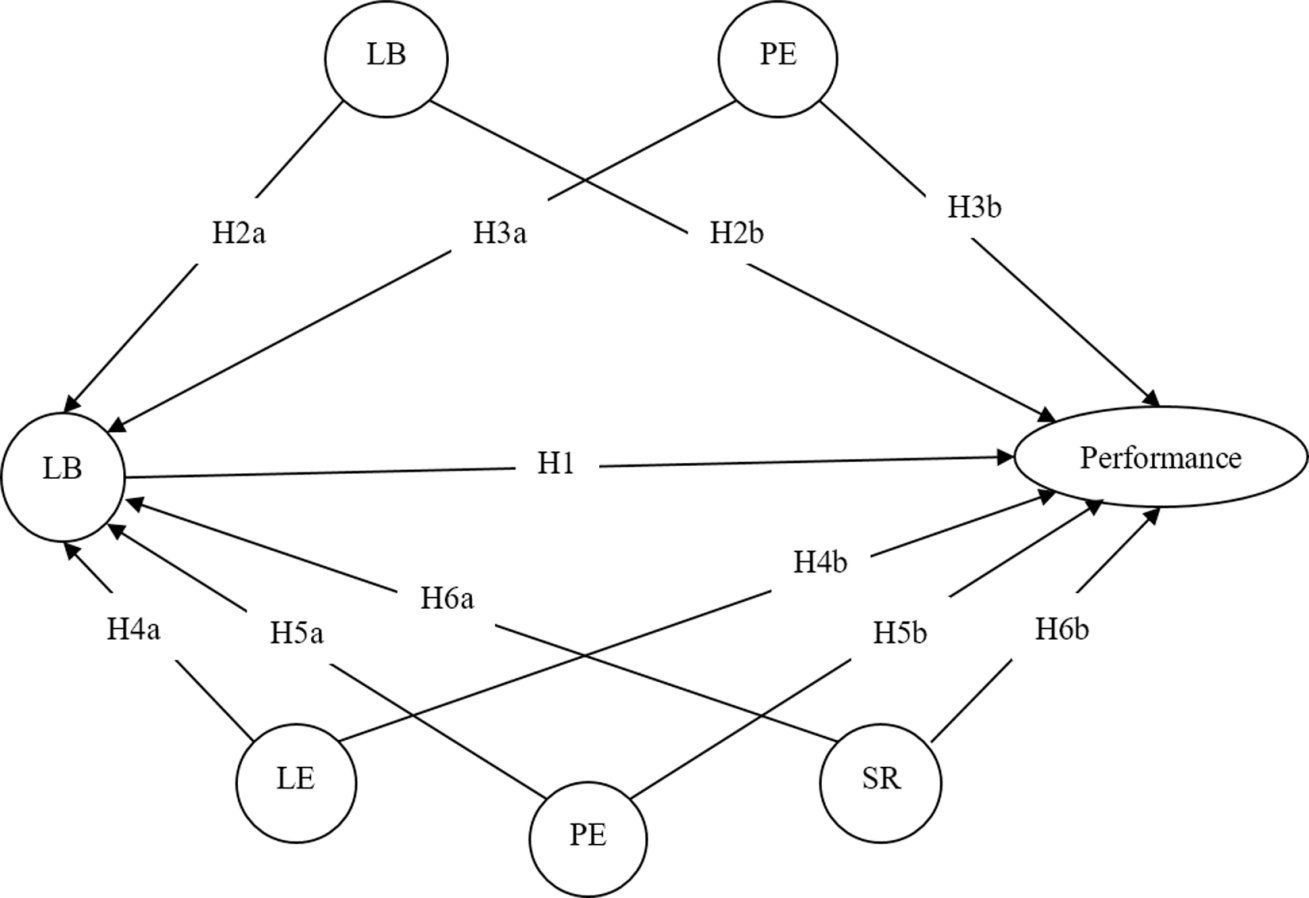
The proposed research model.

Based on the aforementioned assumptions, we employed Partial Least Squares-Structural Equation Modeling (PLS-SEM) to explore and investigate the interrelationships among various influencing factors. PLS-SEM does not presuppose any specific data distribution or sample size requirements [64]. The software of SmartPLS4 was used to validate the developed theoretical model. The evaluation of the formulated research model is executed through a two-phase approach. Initially, the outer measurement model is scrutinized for its validity. Subsequently, the inner structural model is examined to assess its integrity [61]. Fig 2 shows the proposed research model for this study.

The questionnaire for this study was implemented from 26^th^ November 2022 to 10^th^ January 2023, and the survey was completed while organizing MOOC learning. A total of 5,208 responses were gathered. Following the exclusion of questionnaires with brief completion times, missing responses, repetitive answers throughout, and those deemed invalid according to standard criteria, 4,282 questionnaires were deemed valid, representing an effectiveness rate of 82.22%. The basic demographic information collected included the respondents’ gender, major and grade which is presented in Table 4.

**Table 4:** Demographic information of participants in phase 2.

Category	Group	N	%
Gender	Male	907	21.18
	Female	3,375	78.82
Major	Accounting with Big Data	368	8.59
	E-commerce	308	7.19
	Computer network Technology	271	6.33
	Accounting	210	4.90
	Marketing	209	4.88
	Digital media technology	161	3.76
	Online News and Communication	160	3.74
	Internet of Application Technology	151	3.53
	Business management	125	2.92
	Tourism management	116	2.71
	Big data technology	113	2.64
	Pastry Craftsmanship	113	2.64
	E-commerce (3+2)	111	2.59
	Modern logistics management	100	2.34
	others	1,766	41.20
Grade	Freshman	1,430	33.40
	Sophomore	1,813	42.34
	Junior	1,039	24.26
Total		4,282	100

#### 3.3.2 Ethical considerations.

The study was approved by the Academic Research Committee of Chongqing Business Vocational College. Prior to the completion of the online questionnaire by the students, they were made aware of the study’s objectives. Informed consent was obtained from all participants involved in the study. The consent was in the form of written, ensuring that participants were fully informed about the study’s purpose, procedures, potential risks, benefits, and their rights. Throughout the data gathering process and the subsequent reporting of findings in this research, the students’ identities were kept anonymous and confidential with the utmost rigor, safeguarding the privacy of their responses.

#### 3.3.3 Instrument.

The questionnaire survey was developed by the authors for this study with references from previous studies. We randomly selected 242 students for questionnaire pretest. Then we analyzed the results of Cronbach’s α, Pearson’s correlation coefficient, Kaiser–Mayer–Olkin (KMO) and exploratory factor analysis for validity and reliability test. By removing the items that did not meet the requirements, we obtained the final questionnaire comprising items to measure each of five constructs: Learning Motivation (3 items), Perceived Value (3 items), Learning Environment (4 items), Previous Experience (3 items), and Self-regulation (4 items). These constructs were measured using a 5-point Likert scale with “1 = strongly disagree” and “5 = strongly agree”. Data on Learning Behavior and MOOC performance were obtained directly from the MOOC platform. The Learning Behavior consists of 3 items: learning task completion rate, video viewing completion rate and chapter test completion rate. MOOC performance was represented by the final scores in MOOC. The constructs were reviewed by three experts in MOOC for the validity of content. Each expert was asked to indicate their level of agreement with the items and to provide comments and suggestions. The evaluation focused on the adaptability and significance of the items along with their foundational elements, as well as the consensus-driven examination of these items. The factors and their descriptions are shown in Table 5.

**Table 5:** Factor codes and descriptions.

Factor	Code	Description	Sources
Learning Motivation	LM1	I am very interested in MOOC learning.	[8,65]
	LM2	I can learn something new and useful from MOOC.	
	LM3	My teacher asked me to study MOOC.	
Perceived Value	PV1	MOOC learning is helpful for self-improvement.	[4,8,9]
	PV2	MOOC learning can broaden my professional horizons.	
	PV3	I can learn more professional knowledge through MOOC.	
Learning Environment	LE1	I usually study in a place where I can stay focused.	[9,65,66]
	LE2	I always study MOOCs in a fixed place.	
	LE3	I have a device suitable for MOOC learning.	
	LE4	I am very familiar with the equipment for learning MOOCs.	
Previous Experience	PE1	I have participated in MOOC learning before.	[65,67]
	PE2	I have my own MOOC learning method.	
	PE3	I have achieved satisfactory MOOC learning results.	
Self-regulation	SR1	I have set goals for this MOOC study.	[7,65,66]
	SR2	In order to complete the MOOC study, I have made plans in advance.	
	SR3	I will plan carefully and make full use of my study time.	
	SR4	I will learn MOOC step by step instead of waiting until the last minute.	

#### 3.3.4 Data analysis procedure and techniques.

This study used the PLS-SEM based on the analysis of principle components and the SmartPLS4 was used, following the steps below: (a) in the internal consistency analysis, calculations were performed for the Cronbach’s alpha, Composite Reliability (CR), and item load factors. (b) for assessing convergent validity, the average variance extracted (AVE) was determined. (c) discriminant validity was analyzed using the Fornell-Larcker criterion, heterotrait-monotrait (HTMT) ratios, and cross-loadings. (d) the structural model evaluation involved testing the determination coefficient R^2^, path coefficients, and the root mean square residual [61].

## 4. Results

### 4.1 Results from phase 1

**Fig 3:**
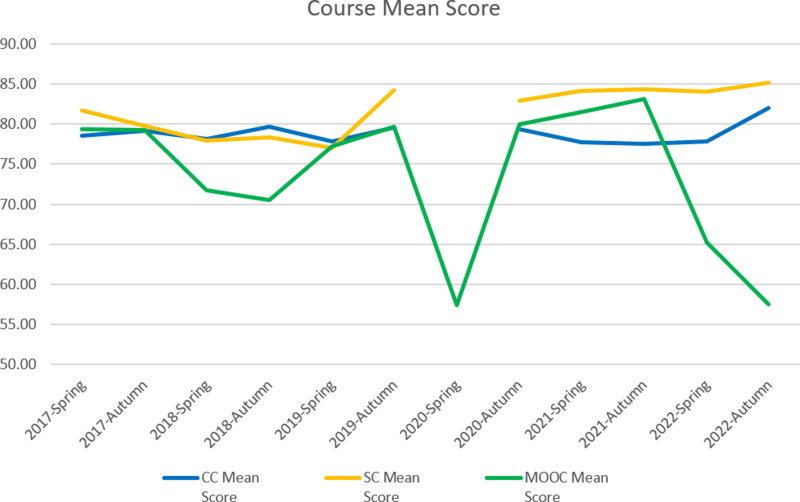
Line chart of course mean score for CC, SC and MOOC.

**Fig 4:**
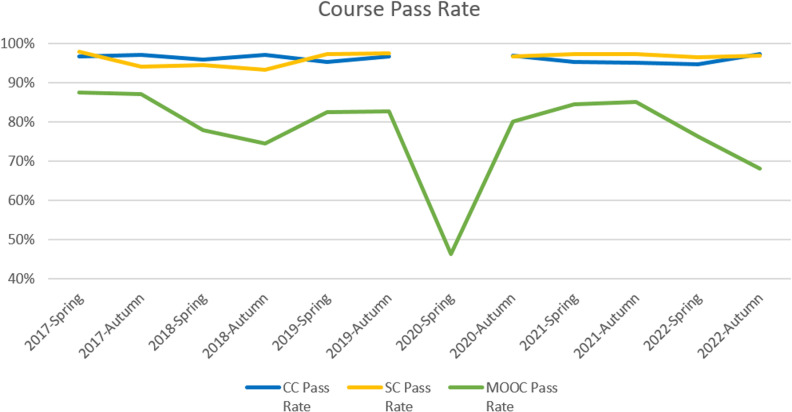
Line chart of pass rate for CC, SC and MOOC.

The results of phase 1 indicated a complicated relationship in terms of mean score and pass rate among CC, SC and MOOC as presented in Table 6, Figs 3 and 4. It is worth noting the data in the semester 2020-Spring. Due to the social distancing policy during COVID-19, the college switched to online learning instead of on-campus teaching. Students could select two MOOC instead of one MOOC and one SC. To help students learn more about pandemic prevention, the college administrator added a MOOC, called “Win the Battle of Pandemic”, for all students. CC were taught online which could not be labled as a traditional course, then we marked them as N/A together with SC in that semester.

**Table 6:** Mean score and pass rate of CC, SC and MOOC by semester.

Semester	CC				SC				MOOC			
	N	Mean Score	Std	Pass Rate	N	Mean Score	Std	Pass Rate	N	Mean Score	Std	Pass Rate
2017-Spring	69,672	78.59	12.79	97%	2,876	81.64	14.31	98%	2,868	79.37	25.07	88%
2017-Autumn	88,146	79.11	12.47	97%	2,842	79.71	20.30	94%	2,565	79.30	25.29	87%
2018-Spring	69,466	78.09	13.70	96%	4,584	77.96	19.50	94%	7,422	71.72	30.56	78%
2018-Autumn	99,369	79.66	13.00	97%	3,215	78.30	21.25	93%	4,446	70.54	31.13	74%
2019-Spring	81,649	77.78	15.45	95%	11,320	76.98	15.07	97%	5,367	77.23	32.23	82%
2019-Autumn	122,732	79.56	13.38	97%	3,322	84.21	14.75	97%	5,111	79.64	33.45	83%
2020-Spring	N/A	N/A	N/A	N/A	N/A	N/A	N/A	N/A	24,786	57.43	40.73	46%
2020-Autumn	128,967	79.31	12.98	97%	3,859	82.92	16.26	97%	6,732	79.97	34.46	80%
2021-Spring	111,477	77.77	15.43	95%	8,786	84.12	15.14	97%	7,406	81.47	28.60	84%
2021-Autumn	149,432	77.49	16.00	95%	4,520	84.35	15.04	97%	11,503	83.10	31.68	85%
2022-Spring	118,258	77.83	16.67	95%	8,479	83.98	17.00	96%	9,168	65.20	29.77	76%
2022-Autumn	146,965	82.04	14.09	97%	5,511	85.16	15.40	97%	5,208	57.53	28.27	68%

*Note:* CC and SC were not available on campus because of COVID-19 2020, and they were marked as N/A.

CC and SC reported higher pass rates than MOOC, and the difference between the MOOC and traditional courses was statistically significant. SC and CC did not have a statistically significant difference with the p-value of 0.876. In terms of course mean score, SC was significantly higher than MOOC and CC at 0.05 level but we did not find a significant difference between CC and MOOC. Paired t-test and Wilcoxon signed ranks test results for comparisons are presented in Tables 7 and 8.

**Table 7:** The paired T-test results for course pass rate and mean score.

Outcome	Group		Mean	SD	Difference	95% CI		t	p-value
Pass Rate	paired 1	CC	96.13%	0.92%	15.63%	11.46%	19.80%	836.03%	0.000
		MOOC	80.50%	5.94%					
	paired 2	SC	96.23%	1.58%	15.73%	11.86%	19.60%	906.70%	0.000
		MOOC	80.50%	5.94%					
	paired 3	SC	96.23%	1.58%	0.10%	−1.29%	1.49%	16.02%	0.876
		CC	96.13%	0.92%					
Mean Score	paired 1	SC	81.76	2.99	2.92	0.92	4.91	3.26	0.009
		CC	78.84	1.32					

**Table 8:** The Wilcoxon Signed Ranks Test for mean score.

	CC-MOOC	SC-MOOC
Z	0.711	2.845
Asymp. Sig. (2-tailed)	0.477	0.004

### 4.2 Results from phase 2

#### 4.2.1 Internal consistency.

Cronbach’s alpha and CR assess how well a set of items “hang together” to measure a single construct. High internal consistency suggests that the items are measuring the same thing, which is a fundamental requirement for a reliable scale [9]. Table 9 displays the factor loadings for the items, along with the CR for each derived factor and their Cronbach alpha coefficients, all of which exceed the suggested threshold of 0.7 [61]. On this basis, construct reliability was established.

**Table 9:** Results of internal consistency.

Item	loading	Cronbach’s alpha	rho_a	rho_c
LB1	0.748	0.846	0.881	0.908
LB2	0.943			
LB3	0.926			
LM1	0.948	0.911	0.912	0.944
LM2	0.910			
LM3	0.905			
PV1	0.902	0.891	0.895	0.933
PV2	0.945			
PV3	0.871			
LE1	0.918	0.816	0.844	0.880
LE2	0.763			
LE3	0.764			
LE4	0.763			
PE1	0.920	0.911	0.917	0.944
PE2	0.951			
PE3	0.892			
SR1	0.843	0.876	0.879	0.915
SR2	0.908			
SR3	0.831			
SR4	0.833			

#### 4.2.2 Convergent validity.

Convergent validity is important because it enhances the trustworthiness of research by ensuring that different measures of the same construct are consistent with each other [68]. Table 10 displays the AVE coefficients for the factors. All factors have AVE values exceeding 0.50, indicating that the factors account for more than half of the variance [61], ensuring the questionnaire’s ability to capture what it intends to measure.

**Table 10:** Results of convergent validity.

Factor	AVE
Learning Behavior	0.769
Learning Motivation	0.849
Perceived Value	0.822
Learning Environment	0.648
Previous Experience	0.849
Self-regulation	0.730

#### 4.2.3 Discriminant validity.

Discriminant validity helps demonstrate that a measure is distinct from other constructs it theoretically should not be related to [69], which strengthens the overall model’s integrity and robustness. The discriminant validity was assessed using the Fornell-Larker criterion and the Heterotrait-Monotrait ratio [61]. Table 11 shows the results of the Fornell-Larker criterion test in which the square roots of all AVEs are greater than their correlations with other constructs.

**Table 11:** Results of discriminant validity (Fornell-Larker).

Factor	Learning Behavior	Learning Environment	Learning Motivation	Previous Experience	Perceived Value	Self-regulation
Learning Behavior	0.877					
Learning Environment	0.586	0.805				
Learning Motivation	0.598	0.613	0.921			
Previous Experience	0.382	0.350	0.377	0.921		
Perceived Value	0.656	0.557	0.582	0.359	0.907	
Self-regulation	0.599	0.553	0.634	0.351	0.556	0.854

Table 12 shows the HTMT ratio results, in which the value of each construct does not exceed the threshold value of 0.85, hence establishing discriminant validity [70]. Therefore, it would be proper to proceed with the evaluation of the structural model.

**Table 12:** Results of discriminant validity (HTMT).

Factor	Learning Behavior	Learning Environment	Learning Motivation	Previous Experience	Perceived Value	Self-regulation
Learning Behavior						
Learning Environment	0.693					
Learning Motivation	0.686	0.699				
Previous Experience	0.432	0.400	0.413			
Perceived Value	0.743	0.645	0.644	0.396		
Self-regulation	0.687	0.645	0.707	0.391	0.625	

#### 4.2.4 Evaluation of the structural model.

**Fig 5:**
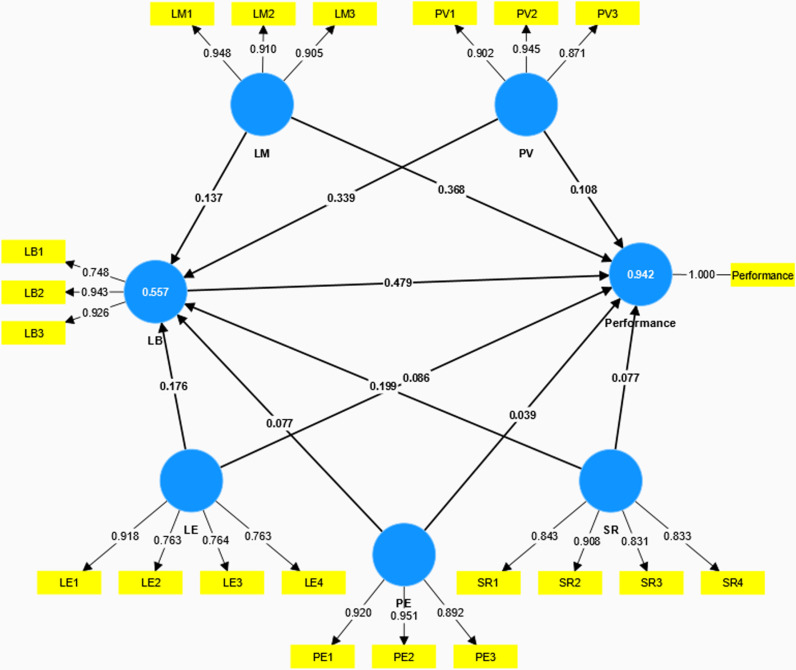
PLS algorithm results.

To clarify the significance of the correlations between the factors, reliance on the model’s determination coefficient (R²) and the analysis of path coefficients is necessary. By examining the information presented in Fig 5, we can find that the latent factors covered by the model have an explanatory power of 55.7% for the variation in learning behavior, and an even higher explanatory power of 94.2% for the variation in MOOC performance. These percentages are measures of how well the model’s latent factors can predict the differences in how individuals engage with the learning process and perform in MOOC settings. This insight is valuable for educators, course designers, and learners alike, as it can inform the development of more effective learning strategies and the enhancement of MOOC platforms to better support student success.

Table 13 presents the path coefficients of the confirmed hypotheses, the significance levels of these relationships, and their respective effect sizes. Hypothesis 1 (H1) determines whether the Learning Behavior significantly affects the MOOC performance. The connection between the two factors is statistically significant, with a reported t-value of 62.927 (β = 0.479, p-value < .001). Therefore, H1 was supported. Furthermore, LM, PV, LE, PE and SR had positively predicted LB thus supporting H2a (β =  0.137, t =  12.863), H3a (β =  0.339, t =  20.294), H4a (β =  0.176, t =  13.114), H5a (β =  0.077, t =  8.997), and H6a (β =  0.199, t =  14.494). LM, PV, LE, PE and SR were found to positively affect the MOOC performance. Therefore, H2b (β =  0.368, t =  58.908), H3b (β =  0.108, t =  15.014), H4b (β =  0.086, t =  15.512), H5b (β =  0.039, t =  9.880), and H6b (β =  0.077, t =  13.704) were also supported. The significant path coefficients and effect sizes across the hypotheses suggest that a multifaceted approach to course design is necessary. Teachers and administrators should consider a holistic view of the learning process, addressing not just content delivery but also the motivational, experiential, and social aspects of MOOC learning.

**Table 13:** Path weights of each hypothesis.

Hypothesis		Path coefficient	T value	p value	f-square
H1	LB -> Performance	0.479	62.927	0.000	1.766
H2a	LM -> LB	0.137	12.863	0.000	0.020
H2b	LM -> Performance	0.368	58.908	0.000	1.068
H3a	PV -> LB	0.339	20.294	0.000	0.145
H3b	PV -> Performance	0.108	15.014	0.000	0.098
H4a	LE -> LB	0.176	13.114	0.000	0.038
H4b	LE -> Performance	0.086	15.512	0.000	0.066
H5a	PE -> LB	0.077	8.997	0.000	0.011
H5b	PE -> Performance	0.039	9.880	0.000	0.021
H6a	SR -> LB	0.199	14.494	0.000	0.047
H6b	SR -> Performance	0.077	13.704	0.000	0.051

To evaluate the magnitude of the relationship between the factors, the effect size measure (f^2^) is employed. The calculation of f^2^ is based on the following interpretation: a value of 0.02 or below is considered a small effect, a value around 0.15 indicates a medium effect, and a value above 0.35 signifies a large effect [61]. The PLS-SEM analysis yielded f^2^ values that spanned from 0.011 to 1.766, with the specific effect sizes for each relationship. Concurrently, the SRMR criterion resulted in a coefficient of 0.048, which is below the threshold of 0.08 suggested by Hu and Bentler [71].

## 5. Discussion

This study compares the differences between MOOC and traditional course in terms of mean score and pass rate through data analysis of the MOOC adoption in a college over a period of six years. Through the research results, it can be found that when MOOC is applied to higher education as a special course type, the outcomes of MOOC are very different from those of existing research. In addition, we verified the proposed influencing factors by constructing the PLS-SEM structural equation model. The results highlight that the proposed factors do have significant impacts on the MOOC performance.

**Q1 Comparing with traditional courses, what are the outcomes of MOOC adoption in higher education?**

The results of the first phase indicate that: 1) MOOC has a lower pass rate than traditional course including CC and SC; 2) MOOC has a lower mean score than SC only; 3) we did not find significant difference between MOOC and CC on the mean score.

After a comparative analysis of data in the past six years, it is found that the pass rate of MOOC is significantly lower than that of CC and SC. This finding is in line with those of [20,21,72] who found the similar results. It can be explained by the differences between MOOC and traditional course. First, teaching methods are different in MOOC and traditional course. Traditional course is taught by the college instructor, with fixed lesson plan and attendance check. MOOC, on the other hand, is entirely self-directed by student. Although MOOC has a deadline for learning, few students will make a study plan [28]. Albelbisi, Al-Adwan [66] and [73] have found that whether students have sufficient self-regulation ability affects the final learning performance of MOOC. The second difference between MOOC and traditional course is the learning environment, including learning device and learning location. SC is taught in classrooms, and the instructor provides the necessary learning facilities, such as computers, operating tools, etc.. MOOC, on the other hand, requires students to purchase their own learning equipment, such as mobile phones, computers, etc. The problem is that not all students have suitable learning equipment. In addition, there is no fixed location for MOOC learning. Therefore, some students choose to study in free classrooms, and some students study in the library or dormitory. The study results from Kobicheva, Tokareva [67] have shown that the learning environment has an impact on the learning outcomes.

We have observed that, over the past six years, the pass rates for MOOC have varied between 68% and 88%. It is important to note that the pass rate for the 2020-Spring semester has been excluded from this calculation, as the college required all students to take a specific course, which deviates from the voluntary nature characteristic of MOOC. These pass rates significantly exceed the 10% completion rate reported in previous studies, such as those by Liyanagunawardena, Adams [74]. This can be explained from the following aspects: First, the MOOC participants in most previous studies were public learners, and their educational levels were uneven. The participants in this study were college students, and their education level and comprehensive quality were generally higher. Huang, Jew [3] have found that learners’ educational level is related to their ability to complete MOOC. Second, the motivations for MOOC learning are different. Public learners mainly choose MOOC according to their own interests and hobbies, which are intrinsic motivations. College students have their own interests in learning MOOC as well as the requirements from the college, which can enhance their motivations. Lee and Song [37] and Huang and Jew [28] have found that motivation has an impact on MOOC learning. Third, the main task of college students is to study, and there are fewer factors that are distracted. The comparison of MOOC for public and college students is shown in Table 14. Therefore, it is easier for college students to stick to completing MOOC studies.

**Table 14:** Comparison of MOOC for public and MOOC in higher education.

Dimension	MOOC for public	MOOC in higher education
Participant	learners with different background	college students with similar background
Motivation	interests	interests and requirements from college
Pressure	no	from college
Course selection	unlimited	limited by college
Learning environment	complicated	mainly in college
Position	low	middle

The results also indicate that the MOOC has lower mean score than SC. As we discussed above, students can choose the courses they are interested in through MOOC and SC. While MOOC may not match the SC in terms of instructional techniques and educational atmosphere, resulting in a lower pass rate and consequently a lower mean score when compared to SC, some research indicates that incorporating MOOC into the curriculum can enhance academic performance. This improvement is attributed to the integration of MOOC as a supplementary teaching approach rather than treating it as an independent course entity, as observed in this particular study.

Finally, there was no significant difference between MOOC and CC in terms of average scores. Despite the lower pass rate in comparison to CC, the average scores are not significantly different, suggesting that while a greater number of students may not complete the MOOC, those who do finish tend to perform better. This indicates that MOOC learning can be more effective for certain learners, yet the challenge of maintaining engagement and completing the course remains a significant issue.

**Q2 What are the factors influencing the MOOC performance from the perspective of education administration in higher education?**

Various factors were found to affect MOOC performance in different types of education [33,44,73,75]. According to Huang and Jew [28], the MOOC, used as a special type of course in higher education, has its unique characteristics comparing with traditional course. The main purpose of phase 2 is to investigate the factors of learning behavior, learning motivation, perceived value, learning environment, previous experience, and self-regulation affecting MOOC performance in higher education. For this, PLS-SEM was used to examine the relationships of each factor and their influence coefficients on MOOC performance. The results indicate that the model is efficient in explaining the significant factors influencing MOOC performance.

The results show that learning behavior has a significantly positive effect on MOOC performance, which is in line with research from Vitiello, Walk [76] and Flanagan, Majumdar [77]. It can be explained by the following two reasons. First, as a type of online study, learners can access MOOC materials only through interactions, which will be easily stored as a series of learning behaviors. Second, generally a large part of MOOC performance is composed of various learning behaviors. When a student’s learning behavior level is increasing, his or her performance will improve to some extent. It is suggested that college educational management and instructor can directly and significantly improve MOOC performance by paying attention to and intervening learners’ learning behaviors. Data on learning behavior can be obtained from the MOOC platform. Analyzing learning behavior and implementing intervention are effective ways to improve learning performance [78]. Educational technologies, such as learning analytics, adaptive learning systems, etc., can provide learners with personalized learning experiences and support based on learning behavior data [79].

The study also shows that learning motivation, perceived value, learning environment, previous experience, and self-regulation have significant effects on learning behavior and MOOC performance. The findings of factors in perceived value, previous experience, and self-regulation are also in agreement with previous studies by Chen and Chen [41], Jung, Kim [39], Lee, Watson [50] and Makhno, Kireeva [51] which concluded that they have a significant positive relationship with learning behavior and MOOC performance. The elements of learning motivation and learning environment in MOOC diverge significantly from other MOOC learning paradigms. As an optional course, college students possess an inherent drive to engage with MOOC content, coupled with expectations set by institutional policies. Consequently, their engagement with MOOC is propelled by a variety of motivations, encompassing intrinsic interest, substantial extrinsic pressures, and potentially amplified motivational factors [28]. We can find that the completion rate of MOOC in higher education is higher than that of normal MOOC [15]. Even in the absence of intrinsic motivation to pursue MOOCs or when faced with the temptation to quit, college students are often spurred on by external motivations from academic authorities and educators. The learning environment, which encompasses aspects such as the learning location and learning tool, is identified as a pivotal factor influencing MOOC performance. Typically, MOOC platforms offer courses and online learning opportunities but do not provide the necessary internet-connected tools. The execution of MOOC studies is contingent upon the availability of digital devices in appropriate locations. The use of mobile devices and the choice of study locations have been recognized as essential for effective MOOC learning within higher education contexts [28]. Public learners will choose MOOC only when they have the necessary learning environment. However, in this study, college students are required to learn MOOC, regardless of whether they have the suitable learning environment or not. Despite the widespread adoption of electronic devices, there are still many students who lack the appropriate tools to engage in MOOC learning. Consequently, even for the same MOOC, the learning outcomes vary when different learning tools are used in distinct learning environments. To enhance the performance of MOOC, college administrators should take a comprehensive approach to ensure that all the necessary components for a conducive learning environment are effectively established and maintained.

## 6. Implications

The findings drawn from this study carry several key implications for various stakeholders involved in the MOOC education ecosystem. Theoretically, this study contributes to furthering our understanding of MOOC learning in higher education. It is among the early works to analyze the outcomes of MOOC adoption as a type of course and determine the relationship between factors and MOOC performance in the specific context of higher education. Practically, college instructors and administrators can draw upon the results from this study and develop appropriate measures when adopting MOOC in higher education.

The first implication is that college administrators may keep adopting MOOC since it has acceptable outcomes. However, schools should be vigilant when using MOOC on a large scale, because while MOOC shows its advantages such as large scale and abundant resources, they also show shortcomings such as low pass rate and limited learning effect. On MOOC platforms, a wealth of learning data is produced, which allows instructors and college administrators to gain precise insights into students’ learning progress through analysis. With the extensive use of information technology, they can employ statistical and machine learning models to gain a profound understanding of their students’ learning behaviors. When students at risk are identified, it is crucial for instructors and administrators to offer timely support.

Furthermore, college administrators must focus on fostering MOOC learning motivation. By understanding the educational needs of their students, they can select and provide relevant courses on the MOOC platform to stimulate students’ interest. Although students may initially be motivated by external pressures from instructors and administrators, this motivation can wane over time. Therefore, it is recommended that instructors and administrators set requirements and implement regular automated reminders to maintain this external motivational pressure.

Lastly, when offering MOOC, it is imperative for college administrators to ensure the availability of an adequate learning environment, including access to digital devices and suitable study spaces. While it may be challenging for colleges to provide every student with a personal device, establishing well-equipped computer labs with computers, headphones, and high-speed internet access for communal use could serve as a viable solution. This includes providing technical support to troubleshoot any issues that may arise during the course of their studies. In addition, administrators should consider creating designated areas within the campus that are quiet, well-lit, and equipped with the necessary infrastructure such as stable internet connections. These spaces should also be accessible to all students, including those with disabilities, to promote inclusivity.

## 7. Limitations and future works

While the current study has yielded substantial findings, it is not without its limitations, which ought to be taken into account for future investigative endeavors. First, the data were gathered from a single sample of students from a higher education institution in China, implying that the circumstances might vary in other regions or nations. It is essential to approach the generalization of these findings and the conceptual model with caution, given that local cultural and operational nuances can markedly affect the relevance and applicability of the results. This study’s findings are constrained due to the absence of qualitative data, which could have significantly enhanced the depth and nuance of the interpretations drawn from the quantitative results.

In future research endeavors, it is highly recommended to further ascertain the robustness and generalizability of the model by expanding the scope of the study to encompass a diverse range of student samples from various educational institutions. This approach would not only validate the model across different academic settings but also provide a more comprehensive understanding of its applicability and effectiveness across a broader spectrum of educational contexts. By including students from multiple institutions, researchers can gather data that reflects a variety of teaching methodologies, learning environments, and student demographics, which are crucial for establishing the model’s versatility and reliability. In addition, future studies would greatly benefit from incorporating qualitative techniques such as interviews or focus groups. These methods can offer a deeper and more intimate understanding of the subject matter, providing insights that quantitative data alone cannot capture. By employing interviews, researchers can engage directly with participants, allowing for a detailed exploration of their thoughts, feelings, and experiences.

## 8. Conclusions

MOOC serves a crucial function in promoting educational equality and facilitating lifelong learning, and they have become increasingly prevalent in the realm of higher education. Previous studies have shown that MOOC has both the benefits of promoting learning and the shortcomings of low completion rate. However, there is still a lack of research on the outcomes of MOOC adoption in college from the perspective of administration. To fill this gap, this study analyzes the MOOC data comparing with tradition course in terms of mean score and pass rate in the past 6 years. Then, we examined the factors affecting MOOC performance and the proposed research model has been validated using the PLS-SEM. The results of the first phase indicate that: 1) MOOC has a lower pass rate than traditional course including CC and SC; 2) MOOC has a lower mean score than SC only; 3) we did not find significant difference between MOOC and CC on the mean score. These findings could be explained by the differences between MOOC and traditional course in terms of teaching method, learning environment and learn demographics. The results of the second phase indicate that learning behavior, learning motivation, perceived value, learning environment, previous experience, and self-regulation have significant and positive influences on the MOOC performance in higher education. The factor of learning environment, including learning tool and learning location, was proposed for the first time, and it is proved to have a significant impact on learning behavior and MOOC performance. The evidence from these results provides holistic insights which could assist the college administrators and instructors to better understand the factors affecting the MOOC performance in higher education. Finally, this research offers insights that can assist administrators and instructors in enhancing MOOC outcomes when integrating MOOC as a course format within college curricula.

## Supporting information

S1 File CC_12semesters. (XLSX)

S2 File SC_12semesters. (XLSX)

S3 File MOOC_12semesters. (XLSX)

S4 File Participants in phase 1. (XLS)

S5 File Participants in phase 2. (XLS)

S6 File Questionnaire. (XLSX)

S7 File Data for PLS-SEM. (CSV)

S8 File Pass rate figure. (XLSX)

S9 File Mean score figure. (XLSX)
